# (*Z*)-3-Chloro-*N*-[(*Z*)-3-(3-chloro-2-methyl­phenyl­imino)­butan-2-yl­idene]-2-methyl­aniline

**DOI:** 10.1107/S1600536811052044

**Published:** 2011-12-10

**Authors:** Jianchao Yuan, Weibing Xu, Tongjian Mei, Yufeng Liu, Xuehu Wang

**Affiliations:** a Key Laboratory of Eco-Environment-Related Polymer Materials of the Ministry of Education, Key Laboratory of Polymer Materials of Gansu Province, College of Chemistry & Chemical Engineering, Northwest Normal University, Lanzhou 730070, People’s Republic of China

## Abstract

In the title compound, C_18_H_18_Cl_2_N_2_, the complete molecule is generated by the application of *C*_2_ symmetry. The C=N bond has an *E* configuration. The dihedral angle between the benzene ring and the 1,4-diaza­butadiene plane is 66.81 (9)°.

## Related literature

For background to the applications of the olefin polymerization Ni(II)-α-diimine catalysts, see: Johnson *et al.* (1995 ▶); Killian *et al.* (1996 ▶). For the effect of the ligand structure on the activity of the catalyst and properties of the products, see: Popeney & Guan (2010 ▶); Popeney *et al.* (2011 ▶); Yuan *et al.* (2005 ▶). For related structures, see: Kose & McKee (2011 ▶); Wei *et al.* (2011 ▶).
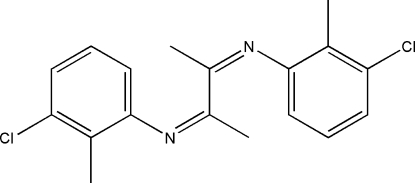


## Experimental

### 

#### Crystal data

C_18_H_18_Cl_2_N_2_
*M* *_r_* = 333.24
Monoclinic, 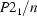

*a* = 8.032 (6) Å
*b* = 7.372 (5) Å
*c* = 14.475 (10) Å
β = 93.533 (7)°
*V* = 855.5 (11) Å^3^
*Z* = 2
Mo *K*α radiation
μ = 0.38 mm^−1^
*T* = 296 K
0.23 × 0.21 × 0.19 mm

#### Data collection

Bruker APEXII CCD diffractometer
Absorption correction: multi-scan (*SADABS*; Bruker, 2008 ▶) *T*_min_ = 0.918, *T*_max_ = 0.932
5279 measured reflections
1588 independent reflections
1199 reflections with *I* > 2σ(*I*)
*R*_int_ = 0.034

#### Refinement

*R*[*F* ^2^ > 2σ(*F* ^2^)] = 0.039
*wR*(*F* ^2^) = 0.115
*S* = 1.06
1588 reflections
102 parameters
H-atom parameters constrained
Δρ_max_ = 0.22 e Å^−3^
Δρ_min_ = −0.28 e Å^−3^

### 

Data collection: *APEX2* (Bruker, 2008 ▶); cell refinement: *SAINT* (Bruker, 2008 ▶); data reduction: *SAINT*; program(s) used to solve structure: *SHELXS97* (Sheldrick, 2008 ▶); program(s) used to refine structure: *SHELXL97* (Sheldrick, 2008 ▶); molecular graphics: *SHELXTL* (Sheldrick, 2008 ▶); software used to prepare material for publication: *SHELXTL*.

## Supplementary Material

Crystal structure: contains datablock(s) I, global. DOI: 10.1107/S1600536811052044/bq2319sup1.cif

Structure factors: contains datablock(s) I. DOI: 10.1107/S1600536811052044/bq2319Isup2.hkl

Supplementary material file. DOI: 10.1107/S1600536811052044/bq2319Isup3.cml

Additional supplementary materials:  crystallographic information; 3D view; checkCIF report

## Figures and Tables

**Fig. 1.**
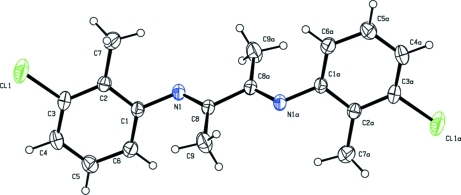
Molecular structure of the title compound, using 30% probability level ellipsoids. Primed atoms are related by the symmetry code (-x + 1, -y + 1, -z).

## supplementary crystallographic information

### Comment

There is a considerable interest in the development of new late transition metal catalysts for the polymerization of α-olefins since Brookhart discovered highly active α-diimine nickel catalysts (Johnson *et al.*, 1995; Killian *et al.*, 1996). It is well known that the ligand structure had significant influence on the product properties and polymerization activities (Popeney & Guan, 2010; Popeney *et al.*, 2011; Yuan *et al.*, 2005). In this study, we designed and synthesized the title compound as a bidentate ligand, and its molecular structure was characterized by X-ray diffraction. In the solid state, the ligand exhibits a *C*_2_ symmetry. The single bond of 1,4-diazabutadiene fragment is (E)-configured. The dihedral angle between the benzene ring and 1,4-diazabutadiene plane is 66.81 (9)°. (Figure 1.) In the crystal packing, there is no hydrogen-bond between the ligand molecules.

### Experimental

Formic acid (0.5 ml) was added to a stirred solution of 2,3-butanedione (0.052 g, 0.6 mmol) and 3-chloro-2-methylaniline (0.0.170 g, 1.2 mmol) in methanol (20 ml). The mixture was refluxed for 24 h, then cooled and the precipitate was separated by filtration. The solid was recrystallized from dichloromethane/cyclohexane (*v*/*v* = 8:1), washed with cold ethanol and dried under vacuum to give the title ligand 0.18 g (90%). Anal. Calcd. for C_18_H_18_Cl_2_N_2_: C, 64.87; H, 5.44; N,8.41; Cl, 21.28. Found: C, 64.97; H, 5.33; N, 8.21; Cl, 21.59. Crystals suitable for X-ray structure determination were grown from a solution of the title compound in a mixture of cyclohexane/dichloromethane (1:2, *v*/*v*).

### Refinement

All hydrogen atoms were placed in calculated positions with C—H distances of 0.93 and 0.96 Å for aryl and methyl type H-atoms. They were included in the refinement in a riding model approximation, respectively. The H-atoms were assigned *U*iso = 1.2 times *U*eq of the aryl C atoms and 1.5 times *U*eq of the methyl C atoms.

### Crystal data

**Table:**

C_18_H_18_Cl_2_N_2_	*F*(000) = 348
*M**_r_* = 333.24	*D*_x_ = 1.294 Mg m^−^^3^
Monoclinic, *P*2_1_/*n*	Mo *K*α radiation, λ = 0.71073 Å
Hall symbol: -P 2yn	Cell parameters from 2237 reflections
*a* = 8.032 (6) Å	θ = 2.8–28.0°
*b* = 7.372 (5) Å	µ = 0.38 mm^−^^1^
*c* = 14.475 (10) Å	*T* = 296 K
β = 93.533 (7)°	Block, colorless
*V* = 855.5 (11) Å^3^	0.23 × 0.21 × 0.19 mm
*Z* = 2	

### Data collection

**Table:**

Bruker APEXII CCD diffractometer	1588 independent reflections
Radiation source: fine-focus sealed tube	1199 reflections with *I* > 2σ(*I*)
graphite	*R*_int_ = 0.034
φ and ω scans	θ_max_ = 25.5°, θ_min_ = 2.8°
Absorption correction: multi-scan (*SADABS*; Bruker, 2008)	*h* = −9→9
*T*_min_ = 0.918, *T*_max_ = 0.932	*k* = −8→8
5279 measured reflections	*l* = −17→17

### Refinement

**Table:**

Refinement on *F*^2^	Primary atom site location: structure-invariant direct methods
Least-squares matrix: full	Secondary atom site location: difference Fourier map
*R*[*F*^2^ > 2σ(*F*^2^)] = 0.039	Hydrogen site location: inferred from neighbouring sites
*wR*(*F*^2^) = 0.115	H-atom parameters constrained
*S* = 1.06	*w* = 1/[σ^2^(*F*_o_^2^) + (0.0428*P*)^2^ + 0.4563*P*] where *P* = (*F*_o_^2^ + 2*F*_c_^2^)/3
1588 reflections	(Δ/σ)_max_ = 0.001
102 parameters	Δρ_max_ = 0.22 e Å^−^^3^
0 restraints	Δρ_min_ = −0.28 e Å^−^^3^

### Special details

**Table:**

Geometry. All e.s.d.'s (except the e.s.d. in the dihedral angle between two l.s. planes) are estimated using the full covariance matrix. The cell e.s.d.'s are taken into account individually in the estimation of e.s.d.'s in distances, angles and torsion angles; correlations between e.s.d.'s in cell parameters are only used when they are defined by crystal symmetry. An approximate (isotropic) treatment of cell e.s.d.'s is used for estimating e.s.d.'s involving l.s. planes.
Refinement. Refinement of *F*^2^ against ALL reflections. The weighted *R*-factor *wR* and goodness of fit *S* are based on *F*^2^, conventional *R*-factors *R* are based on *F*, with *F* set to zero for negative *F*^2^. The threshold expression of *F*^2^ > σ(*F*^2^) is used only for calculating *R*-factors(gt) *etc*. and is not relevant to the choice of reflections for refinement. *R*-factors based on *F*^2^ are statistically about twice as large as those based on *F*, and *R*- factors based on ALL data will be even larger.

### Fractional atomic coordinates and isotropic or equivalent isotropic displacement parameters (Å^2^)

**Table:**

	*x*	*y*	*z*	*U*_iso_*/*U*_eq_	
C1	0.4602 (2)	0.2982 (3)	0.17826 (13)	0.0373 (5)	
C2	0.5314 (2)	0.3432 (3)	0.26633 (13)	0.0382 (5)	
C3	0.5281 (3)	0.2084 (3)	0.33347 (14)	0.0451 (5)	
C4	0.4584 (3)	0.0403 (3)	0.31793 (16)	0.0532 (6)	
H4	0.4589	−0.0453	0.3651	0.064*	
C5	0.3875 (3)	0.0005 (3)	0.23100 (16)	0.0532 (6)	
H5	0.3391	−0.1124	0.2192	0.064*	
C6	0.3887 (3)	0.1286 (3)	0.16168 (15)	0.0469 (5)	
H6	0.3411	0.1013	0.1031	0.056*	
C7	0.6047 (3)	0.5274 (3)	0.28511 (17)	0.0603 (7)	
H7A	0.5640	0.5748	0.3412	0.090*	
H7B	0.5730	0.6070	0.2346	0.090*	
H7C	0.7241	0.5182	0.2916	0.090*	
C8	0.5174 (2)	0.4244 (3)	0.03386 (13)	0.0383 (5)	
C9	0.6332 (4)	0.2775 (3)	0.00740 (18)	0.0672 (8)	
H9A	0.6605	0.2017	0.0601	0.101*	
H9B	0.7334	0.3304	−0.0136	0.101*	
H9C	0.5803	0.2058	−0.0414	0.101*	
Cl1	0.61699 (10)	0.25335 (11)	0.44457 (4)	0.0788 (3)	
N1	0.4462 (2)	0.4357 (2)	0.10980 (11)	0.0421 (4)	

### Atomic displacement parameters (Å^2^)

**Table:**

	*U*^11^	*U*^22^	*U*^33^	*U*^12^	*U*^13^	*U*^23^
C1	0.0400 (11)	0.0413 (12)	0.0309 (10)	0.0048 (9)	0.0052 (8)	0.0042 (8)
C2	0.0360 (10)	0.0453 (12)	0.0334 (11)	0.0021 (9)	0.0046 (8)	0.0013 (9)
C3	0.0424 (12)	0.0634 (15)	0.0295 (11)	0.0035 (10)	0.0009 (8)	0.0075 (10)
C4	0.0594 (14)	0.0556 (15)	0.0455 (13)	−0.0024 (12)	0.0089 (11)	0.0188 (11)
C5	0.0584 (14)	0.0491 (14)	0.0527 (14)	−0.0101 (11)	0.0075 (11)	0.0077 (11)
C6	0.0527 (13)	0.0505 (14)	0.0370 (12)	−0.0034 (10)	0.0001 (9)	−0.0003 (10)
C7	0.0726 (17)	0.0586 (16)	0.0487 (14)	−0.0108 (13)	−0.0031 (12)	−0.0026 (12)
C8	0.0443 (11)	0.0404 (11)	0.0299 (10)	0.0026 (9)	−0.0003 (8)	0.0024 (9)
C9	0.0875 (19)	0.0616 (16)	0.0553 (15)	0.0312 (14)	0.0260 (14)	0.0189 (13)
Cl1	0.0917 (6)	0.1055 (6)	0.0367 (4)	−0.0069 (4)	−0.0154 (3)	0.0111 (3)
N1	0.0523 (11)	0.0433 (10)	0.0308 (9)	0.0050 (8)	0.0021 (7)	0.0058 (8)

### Geometric parameters (Å, °)

**Table:**

C1—C6	1.391 (3)	C6—H6	0.9300
C1—C2	1.404 (3)	C7—H7A	0.9600
C1—N1	1.417 (3)	C7—H7B	0.9600
C2—C3	1.392 (3)	C7—H7C	0.9600
C2—C7	1.498 (3)	C8—N1	1.273 (3)
C3—C4	1.373 (3)	C8—C9	1.493 (3)
C3—Cl1	1.751 (2)	C8—C8^i^	1.500 (4)
C4—C5	1.380 (3)	C9—H9A	0.9600
C4—H4	0.9300	C9—H9B	0.9600
C5—C6	1.378 (3)	C9—H9C	0.9600
C5—H5	0.9300		
			
C6—C1—C2	120.64 (19)	C1—C6—H6	119.6
C6—C1—N1	120.53 (18)	C2—C7—H7A	109.5
C2—C1—N1	118.46 (19)	C2—C7—H7B	109.5
C3—C2—C1	116.2 (2)	H7A—C7—H7B	109.5
C3—C2—C7	123.0 (2)	C2—C7—H7C	109.5
C1—C2—C7	120.82 (19)	H7A—C7—H7C	109.5
C4—C3—C2	123.8 (2)	H7B—C7—H7C	109.5
C4—C3—Cl1	117.42 (17)	N1—C8—C9	126.12 (19)
C2—C3—Cl1	118.82 (18)	N1—C8—C8^i^	116.1 (2)
C3—C4—C5	118.8 (2)	C9—C8—C8^i^	117.8 (2)
C3—C4—H4	120.6	C8—C9—H9A	109.5
C5—C4—H4	120.6	C8—C9—H9B	109.5
C6—C5—C4	119.8 (2)	H9A—C9—H9B	109.5
C6—C5—H5	120.1	C8—C9—H9C	109.5
C4—C5—H5	120.1	H9A—C9—H9C	109.5
C5—C6—C1	120.8 (2)	H9B—C9—H9C	109.5
C5—C6—H6	119.6	C8—N1—C1	122.45 (18)
			
C6—C1—C2—C3	1.1 (3)	Cl1—C3—C4—C5	−179.88 (18)
N1—C1—C2—C3	174.16 (17)	C3—C4—C5—C6	0.4 (3)
C6—C1—C2—C7	−178.5 (2)	C4—C5—C6—C1	−0.2 (3)
N1—C1—C2—C7	−5.4 (3)	C2—C1—C6—C5	−0.6 (3)
C1—C2—C3—C4	−0.9 (3)	N1—C1—C6—C5	−173.5 (2)
C7—C2—C3—C4	178.6 (2)	C9—C8—N1—C1	−4.5 (3)
C1—C2—C3—Cl1	179.13 (14)	C8^i^—C8—N1—C1	176.6 (2)
C7—C2—C3—Cl1	−1.3 (3)	C6—C1—N1—C8	−67.6 (3)
C2—C3—C4—C5	0.2 (4)	C2—C1—N1—C8	119.4 (2)

Symmetry codes: (i) −*x*+1, −*y*+1, −*z*.
